# Keeping Calm and Carrying On: Relating Affect Spin and Pulse to Complex Skill Acquisition and Adaptive Performance

**DOI:** 10.3389/fpsyg.2020.00377

**Published:** 2020-03-10

**Authors:** Kelsey A. Richels, Eric Anthony Day, Ashley G. Jorgensen, Jonathan T. Huck

**Affiliations:** Department of Psychology, University of Oklahoma, Norman, OK, United States

**Keywords:** affect spin, affect pulse, affect variability, acquisition, adaptation, performance, effort

## Abstract

The purpose of this laboratory study involving repeated measures of emotion as 214 undergraduates (58.4% male) learned a complex video game was to address the need for empirical research on dynamic personality constructs by examining how two aspects of affect variability—spin and pulse—explain variance in skill acquisition and adaptive performance. Spin refers to within-person fluctuations in affect pleasantness and activation potential. Pulse refers to within-person fluctuations in affect intensity. Despite research showing high affect variability reflects a personality profile of heighted reactivity to emotionally charged events and poor adjustment, little empirical research has examined their relationships with behavioral outcomes, much less aspects of skilled performance. Compared to traditional measures of personality, which yield weak effects for predicting acquisition and adaptive performance, measures of affect variability hold considerable promise because they, like performance, reflect dynamic within-person phenomena. Accordingly, the main question addressed by this study was whether spin and pulse incrementally explain acquisition and adaptive performance beyond Big Five measures of personality. In general, we expected harmful, incremental effects for both spin and pulse, and hypothesized two mechanisms for these harmful effects: (1) by undermining effort and (2) by undermining the effort-performance relationship. Using a task-change paradigm and discontinuous growth modeling that disentangled adaptation from acquisition, results showed that affect variability, independent of the Big Five, produced harmful effects via both hypothesized mechanisms. Participants higher in affect spin and pulse showed less sustained effort across performance sessions and exhibited lower performance. Furthermore, the harmful effects of spin and pulse were stronger in adaptation compared to acquisition, with pulse showing stronger direct effects on performance during adaptation and spin moderating the effort-performance relationship such that effort was only beneficial during adaptation for those lower in spin. In light of these results, one might question the common advice “keep calm and carry on,” which may not be viable for persons high in affect variability. Accordingly, results are discussed in terms of the need to better understand the specific mediating processes by which high affect variability undermines success across a variety of learning and performance contexts.

## Introduction

Today, the capacity to acquire and adapt skills is more important than everpc. The performance demands of contemporary work environments are becoming increasingly more nuanced, fast-paced, and dynamic (Bell et al., 2017). Occupations are evolving at a rapid pace and becoming more complex and unpredictable (Noe et al., 2014). Learning new tasks and adapting to unexpected changes are the new normal. Accordingly, adaptability is now critical to many contemporary occupations and workplace environments (Pulakos et al., 2000; Ployhart and Bliese, 2006; Baard et al., 2014; Jundt et al., 2014). In addition, technology has made life outside of work more fast-paced and complex. With advanced technology becoming more accessible to the general public, adaptability is becoming a general life skill necessary for successful functioning in the 21st century (Baird and Griffin, 2006).

The growing importance of adaptability prompts an ongoing research question: Are there certain characteristics that make individuals more or less adaptable? By examining affect variability as an aspect of personality distinct from the Big Five (i.e., agreeableness, consciousnesses, extraversion, neuroticism, and openness), the broad aim of the present research was to shed more light on the non-cognitive traits that give rise to people’s capacity to be successful when learning new tasks and experiencing unexpected task changes (Baard et al., 2014). Specifically, we examined the roles played by two aspects of affect variability—spin and pulse—in the context of acquiring and adapting skill on a complex computer game. By tracking affect, effort, and performance using repeated measures before and after a change in task demands, we examined the effects of affect variability as a dynamic personality trait in a way that treated skill acquisition (SA) and adaptation as meaningfully distinct but related processes.

In doing so, the present study addressed a call for empirical research on the role of dynamic personality constructs in explaining adaptive performance, as previous research examining the relationships between personality and adaptive performance has yielded inconsistent and overall weak effects (Huang et al., 2014). Furthermore, this study addresses the popular advice “keep calm and carry on” which reflects the importance of maintaining composure and focus in the face of difficulties. This advice may be easier said than done, and paying it heed is likely a function of personal characteristics.

### Adaptive Performance and the Role of Personality

Although it is common for researchers to examine adaptive performance in terms of a general factor that addresses differences in how individuals handle change (e.g., Pulakos et al., 2000; Huang et al., 2014), scholars have recognized that multiple dimensions likely comprise adaptive performance (e.g., Pulakos et al., 2000). In particular, a two-factor model that distinguishes reactive and proactive forms of adaptation is conceptually useful. Reactive adaptation refers to how individuals handle prescribed demands, whereas proactive adaptation refers to the initiative that individuals take in creating new demands (Ployhart and Bliese, 2006; Berg et al., 2010; Huang et al., 2014). Whether examined in terms of a single overall factor or reactive and proactive forms, Huang et al.’s (2014) meta-analysis revealed overall weak relationships involving personality variables and adaptive performance outcomes.

In general, meta-analytic evidence shows that correlations between traditional self-report measures of personality and task performance tend to be weak, with scores for conscientiousness yielding the strongest among the Big Five factors with corrected coefficients topping around 0.25 and corrected coefficients for the other factors topping around 0.13 (Sackett and Walmsley, 2014). Correlations involving SA and training outcomes are also weak. Although positive indirect effects via more proximal motivation predictors like effort or motivation to learn are likely, direct effects of personality scores tend to be inconsistent, often near zero, and sometimes negative, even for conscientiousness (Colquitt et al., 2000). With respect to adaptive performance, research again shows weak and often inconsistent effects for the Big Five factors (e.g., Griffin and Hesketh, 2003). Huang et al.’s (2014) meta-analysis of facets of emotional stability, extraversion, and openness suggested that emotional stability is the most important personality contributor to reactive forms of adaptive performance, extraversion is the most important personality contributor to proactive forms of adaptive performance, and openness does not contribute to adaptive performance. With this pattern of effects in mind, Huang et al. (2014) concluded that adaptive performance is more successful for those who desire status and power yet remain calm and even-tempered. Nevertheless, the effects in Huang et al.’s (2014) meta-analysis were weak (corrected, operational validities ranged from 0.00 to 0.20) and point to the importance of moving beyond traditional self-reports of the Big Five in relation to the dynamics associated with proximal mediating mechanisms (Matthews, 2018).

To our knowledge, no empirical research has taken a temporal perspective when examining the contributions of personality to SA and adaptive performance. Learning, both acquisition and adaptation, is by nature temporal and dynamic. Cross-sectional research, even predictive designs, are unable to capture acquisition and adaptation as related yet meaningfully distinct dynamic processes. Fluctuations in cognition, affect, and behavior are inherent in learning (Sitzmann and Weinhardt, 2015). Notions of affect variability speak to how repeated measures of affect can be used to capture important between-person differences in within-person fluctuations over time. That is, the experience of emotions over time differs across individuals. Given a similar performance context, some individuals will experience more stability in their emotions while others may experience more volatility. Traditional measures of the Big Five, even measures of emotional stability, do not adequately speak to the dynamic nature of human phenomena (Fleeson and Jayawickreme, 2015). In this way, the present research reflects a basic premise of Whole Trait Theory: there are fluctuations in the expressions of traits, even the expression of the Big Five, which traditionally have been thought to be fairly stable (Fleeson and Jayawickreme, 2015). Specifically, affect spin and pulse are unique aspects of personality that provide a more nuanced understanding of how fluctuations in affect relate to behavior that can speak to the glib advice “keep calm and carry on.”

### Affect Spin and Pulse

Spin and pulse are two aspects of personality that speak to the intraindividual variability in affect experienced across time and circumstances (Moskowitz and Zuroff, 2004; Kuppens et al., 2007). *Affect spin* refers to variability in the pleasantness and activation potential of affective states, and *affect pulse* refers to variability in the intensity of affective states irrespective of their pleasantness and activation potential (Moskowitz and Zuroff, 2004; Kuppens et al., 2007).

Empirical support for affect spin and pulse as meaningful personality traits has been shown in several ways. Although there is no universally agreed upon definition of a personality trait (Augustine and Larsen, 2012), temporal stability of scores is an important psychometric property of scales, and a plethora of longitudinal research has established the test-retest reliability of personality scores, including measures of the Big Five (Conley, 1985). Likewise, research has established the temporal stability of spin and pulse (Moskowitz and Zuroff, 2004). Research has also shown that while affect variability does show convergent validity with some aspects of personality, affect variability is sufficiently distinct to be considered unique from the Big Five (Eid and Diener, 1999). For example, mean levels of affect and the Big Five only explain up to 52% of the variance in affect variability (Eid and Diener, 1999). More specifically, previous research has shown that affect spin is lower for those with higher emotional stability, conscientiousness, and extraversion, whereas affect pulse lacks a consistent relationship with any Big Five personality constructs (Kuppens et al., 2007). These differential relationships with personality traits, demonstrated through both correlational and regression analyses, show that affect spin and pulse are meaningfully distinct to warrant separate consideration (Kuppens et al., 2007). However, while relationships with the Big Five and affect spin and pulse have been established, there is a lack of research examining the differential relationships that spin and pulse have with outcome variables in comparison to those of the Big Five. Given that the burgeoning empirical literature on affect variability has focused almost exclusively on spin without much attention to pulse (e.g., Beal et al., 2013; Park, 2015; Clark et al., 2016), an important contribution of the present study is that we comparatively examined the unique relationships of both spin and pulse with task-focused effort and performance.

### Affect Variability and Performance

**FIGURE 1:**
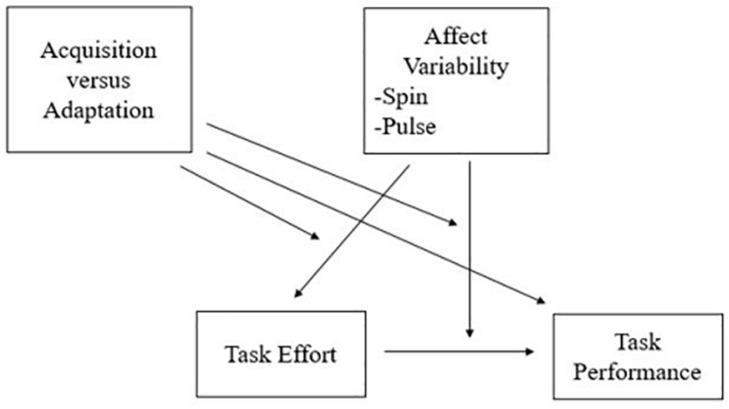
Proposed1pc model of relationship between task performance and task effort, moderated by affect variability and acquisition/adaptation phase.

Although there is a lack of research focused on the relationship between affect variability and behavioral outcomes (Clark et al., 2016), the general profile of those high in affect variability speaks to heightened reactivity to emotionally charged events and difficulty adjusting from such events (Beal and Ghandour, 2011). In the present study, we further the understanding of the distinctiveness of affect spin and pulse in relation to the Big Five by specifically examining their incremental relationships with self-reported effort and objective task performance in the context of both SA and skill adaptation. We tested two mechanisms by which spin and pulse might undermine SA and adaptation. One, spin and pulse may directly influence effort. Two, spin and pulse may moderate the relationship between effort and performance. Figure 1 shows the framework of the mechanisms tested.

This study builds on research showing how effort tends to decrease over the course of learning in relation to diminishing increases in knowledge and skill (Kanfer and Ackerman, 1989; Day et al., 2017; Hardy et al., 2019a). Although much of the decrease in effort can be attributed to ceiling effects—limits to the amount of new knowledge and skill to be gained—some of the decreases are due to the tendency for individuals to satisfice (Simon, 1972) or settle on suboptimal performance strategies (Day et al., 2017; Hardy et al., 2019a). There are a variety of reasons why those high in affect variability may be less likely to sustain their effort over time, some associated with withdrawal from task demands and others from the re-direction of cognitive resources to meet task demands.

Previous research shows that those high in affect spin exhibit a general profile associated with maladjustment. In addition to higher levels of neuroticism and lower levels of agreeableness, those with high affect spin also tend to hold negative expectations for their future (Kuppens et al., 2007). It has been postulated that this negative profile is at least in part due to the emotional swings experienced by those high in affect variability, which make daily life unpredictable (Kuppens et al., 2007). This unpredictability also leads to greater psychological strain as individuals strive to meet performance demands (Beal et al., 2013). Moreover, alongside research showing individual differences in the perception of stressful events (Gunnar and Quevedo, 2007), affect variability plays a role in how individuals perceive performance demands (Beal et al., 2013). Those with high affect variability likely perceive fast-paced, complex performance contexts (such as the one used in this study) as a hindrance stress, with constraints perceived to be outside of one’s control and thus threatening, whereas those with lower affect variability likely view such contexts as a challenge stress, with constraints perceived to be within one’s control and thus an opportunity (Beal and Ghandour, 2011). In contrast to increases in task engagement and goal striving associated with challenge stress, hindrance stress is associated with task withdrawal and burnout (Podsakoff et al., 2007; Giorgi et al., 2017; Deng et al., 2019). Thus, taken together, the negative expectations, unpredictability, and strain associated with high affect variability undermines initial task-focused effort in fast-paced, complex performance contexts and leaves individuals struggling to maintain effort levels over time.

Even in cases when individuals try to persist in meeting task demands, the unpredictability associated with high affect variability can lead to a reduction in task-focused effort as individuals re-direct cognitive resources to regulating their emotional swings (Beal and Ghandour, 2011; Park, 2015). Prior research has linked emotion control to successful acquisition and adaptive performance (Bell and Kozlowski, 2008; cf. Sitzmann and Ely, 2011; Jundt et al., 2014; Niessen and Jimmieson, 2016). Being able to maintain control over both the range and intensity of emotions felt over a period of performance provides more stability to the individual and prevents emotions from shifting one’s focus toward issues outside the task at hand. If an individual feels a broad range of emotions, he/she may feel the need to regulate these emotions, either to reduce negative feelings or to stay within a socially accepted norm of emotion projection. Emotion regulation, specifically when regulating negative emotions, leads to decreases in cognitive functioning (Carver and Scheier, 1981; Richards and Gross, 2000; Richards et al., 2003). Individuals high in spin and pulse therefore require more emotion regulation to meet the demands placed on them, depleting the cognitive resources available for task-related effort. Whether by giving up on meeting task demands or by re-directing cognitive resources to regulating emotions, we would expect less initial and sustained task-focused effort for individuals higher in affect spin and pulse. Therefore, we tested the following hypotheses.

*Hypothesis 1:* Higher levels of (a) affect spin and (b) affect pulse will be associated with lower initial task-focused effort.
*Hypothesis 2:* Higher levels of (a) affect spin and (b) affect pulse will be associated with lower sustained task-focused effort.

To continue to make gains over time, learners must use their cognitive resources to make decisions regarding whether to explore the potential payoffs of new strategies or to exploit and refine existing strategies (Hardy et al., 2019a). However, building upon previous strategies may prove difficult for those high in affect spin and pulse as they experience high strain, considering that high-intensity short-term distress has been shown to disrupt memory (Dhabhar, 2018). Thus, even if effort is put forth, those high in affect spin and pulse may not be able to use this effort to their best ability, as they may be unable to build upon previously gained knowledge. In this vein, the positive effects of effort are less likely to occur for persons high in spin or pulse. Building upon previous strategies may also prove difficult given the inherent emotional fluctuations for those high in affect variability. In the context of SA and adaptation, it is not only important to put forth and sustain effort, but it is also important to direct effort consistently toward learning task demands. If effort is not consistently applied, then it will not yield the development of effective strategies for successful task performance (DeShon and Alexander, 1996). Without emotion control, experiencing a constant flux of emotions likely leads to haphazard learning. Regardless of the magnitude of task-related effort, if effort is directed haphazardly, led by fluctuations in emotion rather than being directed in a systematic way, then it becomes difficult for learners to discover, apply, and fine tune needed performance strategies. Taken together, we tested the following hypothesis.

*Hypothesis 3:* Affect variability will moderate the effects of effort on performance such that the positive effects of effort will be lower for individuals higher on (a) affect spin and (b) affect pulse.

### Affect Spin and Pulse in Adaptation Versus Acquisition

Although no research has linked spin or pulse directly to adaptive performance *per se*, previous research has shown a link between spin and adjustment to negative or emotionally charged events (Beal and Ghandour, 2011). Higher spin is associated with poorer adjustment. However, less research has been devoted to pulse and adjustment. Nevertheless, we posit poorer adjustment for those higher in pulse, because proneness to large fluctuations in emotional intensity likely exacerbates affective reactions to emotionally charged events. Whether unexpected changes in task demands produce a more unpleasant, activating, or intense experience, fluctuations in emotions in general prompt greater distress, strain, emotion regulation, and cognitive withdrawal.

Therefore, we postulate that spin and pulse have stronger effects on effort and performance during skill adaptation compared to acquisition. Over the course of learning, performance becomes less effortful (Kanfer and Ackerman, 1989). However, when changes to task demands occur, the effectiveness of learned strategies is likely disrupted and in turn, additional effort is needed for successful adaptation. Novel aspects of the task must be identified and explored, and previous strategies must be replaced or altered, all of which requires effort. This need to override or adjust previous strategies and develop new strategies is likely to lead to an immediate decrease in performance directly following a task change as well as a slower increase in performance during reacquisition (Lang and Bliese, 2009). This novelty may be particularly challenging for those high in affect variability, given their difficulty adjusting following difficult and emotionally charged events (Beal and Ghandour, 2011).

During adaptation, individuals must not only learn new performance strategies and modify existing performance strategies, but they must also unlearn strategies that are no longer effective—forgoing automated processes. In other words, an important aspect of successful adaptation includes some degree of breaking old habits. This dual process of simultaneously learning and unlearning puts a high premium on task-focused effort. Thus, traits that may have an impact on directing and sustaining effort, like affect spin and pulse, are particularly relevant to adaptive performance. Additionally, this change in demands may lead to higher psychological strain (Giorgi et al., 2017), particularly for those high in spin and pulse, leading to additional deleterious effects on effort and performance during adaptation. Therefore, we tested the following hypotheses.

*Hypothesis 4:* The negative direct effects of (a) affect spin and (b) affect pulse on overall levels of effort will be stronger in adaptation than acquisition.
*Hypothesis 5:* The negative direct effects of (a) affect spin and (b) affect pulse on sustained levels of effort will be stronger in adaptation than acquisition.

As previously discussed, in addition to undermining effort, affect spin and pulse may also moderate the relationship between effort and task performance. Consistent with this perspective, haphazardly applying and revising performance strategies during the adaptation phase should be more harmful to performance than it would during acquisition, as adaptation requires the individual to not only learn a new set of strategies, but also to unlearn previous strategies. Therefore, we tested the following hypothesis.

*Hypothesis 6:* The negative moderation effect of (a) affect spin and (b) affect pulse on the effort-performance relationship will be stronger in adaptation than acquisition.

## Materials and Methods

### Participants

Data from Jorgensen (2017) was used to test the present study’s hypotheses. After receiving Institutional Review Board approval, 232 undergraduate students attending a large public university in the Southwestern U.S. participated in exchange for research credit in a psychology course. Participants were recruited from an online, internal study listing database. Participants were told that they would have an opportunity to receive study credit while playing a computer-based first-person-shooter video game, along with the chance to win a gift card. No restrictions were placed on participants beyond being 18 or older (or obtaining parental permission if under 18) and proficiency in English.

Data from 18 of the participants were removed before analysis due to incomplete data (e.g., due to computer error; *n* = 12), flatlining repeatedly on performance measures (e.g., zero kills and zero deaths; *n* = 4), or failure to follow instructions (e.g., starting a game before instructed; *n* = 2). The removal of this data resulted in a final sample of 214 participants (58.4% male). The age range of participants was from 17 to 32 years (*M* = 19.20, *SD* = 1.70). One hundred thirty-four participants reported their ethnicity as Caucasian (62.6%), 23 as Asian (10.7%), 18 as Hispanic/Latino (8.4%), 14 as African American (6.5%), 12 as Native American (5.6%), 8 as Multiple (two or more ethnicities) (3.7%), and 5 reported as other (2.3%).

### Performance Task

The experimental task used was Unreal Tournament 2004 (UT2004; EPIC Games, 2004), a commercially available first-person shooter computer game that has also been used in previous research on self-regulated learning, complex SA, and adaptive performance (Hughes et al., 2013; Hardy et al., 2014). Although unique as compared to other criterion tasks used in traditional training and SA studies, UT2004 is both relatively easy to learn yet difficult to fully master, providing ample opportunity for growth following a typical SA curve within a 4-h period without the problems of range restriction associated with ceiling effects (Hardy et al., 2019b). In addition, UT2004 reflects the demands of a complex and fast-paced performance environment, and its use as a criterion task is particularly relevant due to the rise of simulation- and game-based training platforms (American Society for Training and Development, 2015) as well as interest in self-regulated learning (Sitzmann and Ely, 2011).

The objective was to destroy computer-controlled opponents (i.e., bots), while minimizing the destruction of one’s own character. Participants could also collect new weapons or resources (i.e., power-ups) during each game to increase their own character’s health or offensive or defensive capabilities. Upon destruction of a participant’s character, that character would reappear in a random location with default weapons and capabilities. The game was “every character for him- or herself,” which means that the computer-controlled bots were competing against each other, as well as the participant’s character. UT2004 involves both cognitive and perceptual-motor demands. Participants used a mouse and a keyboard simultaneously to move and control their character, while also learning the strengths and weaknesses of different strategies and weapons, quickly deciding which to use in specific circumstances.

### Procedure

Participants completed the study at individual computer stations. No more than six individuals participated at the same time. They were told upon entry to the lab that the purpose of the study was to examine how people learn to play a complex, dynamic video game. Participant*s* first completed an informed consent form, followed by a battery of self-report control measures. Participants were told that they would be entered into a performance-based lottery to win one of five, $25 gift cards for each trial in which their score was in the top 50% of all participants for that specific trial. Participants then watched a 15-min training presentation on UT2004 which explained the basic game controls, rules, and power-ups, followed by a 1-min practice trial that was free of competing bots. The purpose of this trial was to allow participants to become familiar with the controls, display, and the game environment without having to deal with any opponents.

Participants then completed 14 sessions, each consisting of two 4-min trials (i.e., 28 trials total). The length of the trials was chosen based on previous research using UT2004 (e.g., Hughes et al., 2013; Hardy et al., 2014, 2019b). Performance across the trials was collapsed into 14 measurement occasions (i.e., an average of the performance across each pair of two trials), as is consistent with previous studies using discontinuous growth curve modeling (e.g., Lang and Bliese, 2009; Niessen and Jimmieson, 2016; Howe, 2019). Collapsing trial scores into session scores allows for more stable estimates of performance by reducing noise (Howe, 2019).

To track fluctuations across time, participants completed self-report measures of state-based affect (PANAS) and effort following each session. Self-report measures were consistent throughout, meaning that breaks between sessions were similar in length (about 2 min), but with additional 4-min rest breaks before Sessions 4 and 11. During the first seven sessions, participants competed against two computer-controlled opponents which were set to a difficulty level of 4 (on a 1-to-8 scale). Changes in task demands, designed to prompt reactive adaptation, occurred following the seventh session (i.e., the halfway point) without any warning, increasing the task complexity (Hughes et al., 2013). During these sessions, players competed against nine computer-controlled opponents at a difficulty setting of 5. Additionally, the game environment (i.e., the game map) was much bigger, with wider spaces, multiple levels of platforms, and edges. The edges allowed players to fall over the end of the map, leading to their own self-destruction. The game characteristics for the pre- and post-change trials were the similar to those used by Hardy et al. (2014) to measure analogical and adaptive transfer performance, respectively. Following the 14th session, participants were debriefed.

### Measures

#### Control Variables

Self-report ACT/SAT scores were used as a measure of general mental ability (GMA). SAT scores were converted to the ACT scale.

Given first-person shooter video games have been shown to yield gender differences regarding performance and enjoyment levels (Hopp and Fisher, 2017) and history of playing these games (Hartmann and Klimmt, 2006), we chose to control for both gender and video game experience. Gender was measured using a self-report.

As a proxy for pre-training video game knowledge, prior video game experience was measured using a 4-item scale. The first two questions were: (a) *“Over the last 12 months, how frequently have you typically played video/computer games?”* (*M* = 2.92, *SD* = 1.42) and (b) *“Over the last 12 months, how frequently have you typically played first-person shooter video/computer games (e.g., Call of Duty, Half-Life, Halo, Unreal Tournament)?”* (*M* = 2.35, *SD* = 1.33). These questions were measured using a 5-point Likert scale (1 = *not at all*, 2 = *rarely or just a few times*, 3 = *monthly*, 4 = *weekly*, 5 = *daily*). The second two items asked how many hours per week participants play (a) any type of video/computer game (*M* = 4.61, *SD* = 6.59, min. = 0.00, max. = 35) and (b) specifically first-person shooter video/computer games (*M* = 2.03, *SD* = 4.03; min. = 0.00, max. = 30). The scores for each of the pairs of items were standardized, and then averaged into an overall standardized composite score.

The Big Five personality dimensions were also used as control variables to examine the independent effects of affect variability. The Big Five were measured using Goldberg’s 100 Unipolar Markers (Goldberg, 1992). Using a 9-point Likert-type scale (1 = *extremely inaccurate*, 9 = *extremely accurate*), participants rated a list of 100 common human traits in terms of how accurately the traits described the participant him- or herself. Each of the five factors consisted of 20 items, with a scale score for each factor consisting of the average of their respective item ratings.

#### Affect Variability

Scores for spin and pulse were based on responses to a 16-item version of the Positive and Negative Affect Schedule that was adapted for the context of this study (PANAS; Watson et al., 1988). Following each session, participants were instructed to answer according to how they felt during the previous two trials, responding on a 9-point Likert-scale after each session (1 = *very slight/not at all*, 3 = *a little*, 5 = *moderately*, 7 = *quite a bit*, 9 = *extremely*). The scale measured four different areas of affect using 16 different emotions that varied with respect to valence and activation potential (i.e., arousal). The adjectives *enthusiastic*, *excited*, and *happy* were used to assess positive activating (PA) emotions. The adjectives *at ease*, *calm*, and *relaxed* were used to assess positive deactivating (PD) emotions. The adjectives *angry*, *anxious*, *frustrated*, *irritated*, *tense*, and *uneasy* were used to assess negative activating (NA) emotions. The emotions *bored*, *disappointed*, *discouraged*, and *fatigued* were used to assess negative deactivating (ND) emotions.

Before beginning calculations for affect spin and pulse, valence and activation scores were calculated for each participant for each session of assessment. Valence is calculated as (PA + PD) − (NA + ND) (Kuppens et al., 2007). Activation is calculated as (PA + NA) − (PD + ND) (Kuppens et al., 2007). Mean valence and activation scores were then calculated, and standard deviations of the repeated scores were used to calculate valence variability (i.e., the standard deviation of pleasure-displeasure that occurs within person) and activation variability (i.e., the standard deviation of activation-deactivation that occurs within person), which were used as control variables, consistent with previous research (Park, 2015). While valence variability and activation variability both describe fluctuations of emotions across time, they do so in a uni-dimensional way (Park, 2015), as compared to affect spin and pulse, which are more representative measures of affective changes that capture both valence and activation within one measure (Beal et al., 2013).

##### Affect spin

*Affect spin* was calculated based on the framework provided by Moskowitz and Zuroff (2004) and following the procedure of Kuppens et al. (2007). Spin, defined as “the circular standard deviation of responses,” represents how much a participant moves “between different angles in the core affect space” (Kuppens et al., 2007, p. 266). Calculations began by finding the unit vector for each session.

$$\left(\frac{v\,a\,l\,\text{e}\,n\,c\,e_{t}}{\sqrt{v\,a\,l\,e\,n\,c\,e_{t}^{2}+a\,c\,t\,i\,v\,a\,t\,i\,o\,n_{t}^{2}}},\frac{a\,c\,t\,i\,v\,a\,t\,i\,o\,n_{t}}{\sqrt{v\,a\,l\,e\,n\,c\,e_{t}^{2}+a\,c\,t\,i\,v\,a\,t\,i\,o\,n_{t}^{2}}}\right)$$

Next, the vector of all observations for one given participant, *R*, was calculated as follows.

$$\left(\sum_{\text{t}=1}^{\text{n}}\frac{v\,a\,l\,\text{e}\,n\,c\,e_{t}}{\sqrt{v\,a\,l\,e\,n\,c\,e_{t}^{2}+a\,c\,t\,i\,v\,a\,t\,i\,o\,n_{t}^{2}}},\sum_{\text{t}=1}^{\text{n}}\frac{a\,c\,t\,i\,v\,a\,t\,i\,o\,n_{t}}{\sqrt{v\,a\,l\,e\,n\,c\,e_{t}^{2}+a\,c\,t\,i\,v\,a\,t\,i\,o\,n_{t}^{2}}}\right)$$

The length of *R* was then calculated as

$$\sqrt{\frac{\sum_{\text{t}=1}^{\text{n}}\,\frac{v\,a\,l\,e\,n\,c\,e_{t}}{v\,a\,l\,e\,n\,c\,e_{t}^{2}+a\,c\,t\,i\,v\,a\,t\,i\,o\,n_{t}^{2}}+\sum_{\text{t}=1}^{\text{n}}\,\frac{a\,c\,t\,i\,v\,a\,t\,i\,o\,n_{t}}{v\,a\,l\,e\,n\,c\,e_{t}^{2}+a\,c\,t\,i\,v\,a\,t\,i\,o\,n_{t}^{2}}}{n}}$$

The length of $R\,\left(\frac{||\vec{R}||}{n}\right)$ can range from 0 to 1. If there is no variability in the angles, then $\frac{||\vec{R}||}{n}$ will equal 1. If the angles are dispersed widely enough to cancel each other out, then $\frac{||\vec{R}||}{n}$ approaches 0 (Kuppens et al., 2007). The final calculation of spin involves the standard deviation of the angles of the unit vectors, which is calculated as

$$\sqrt{-2\,l\,n\,\left(\frac{||\vec{R}||}{n}\right)}$$

This is final calculation of affect spin may range from 0 to infinity (Kuppens et al., 2007).

##### Affect pulse

*Affect pulse* was also calculated based on the framework provided by Moskowitz and Zuroff (2004) and following the procedure of Kuppens et al. (2007). Pulse, the “within-person standard deviation of the distances” between reports of emotions (Kuppens et al., 2007, p. 266), was calculated as

$$\sqrt{v\,a\,l\,e\,n\,c\,e_{t}^{2}+a\,c\,t\,i\,v\,a\,t\,i\,o\,n_{t}^{2}}.$$

#### Effort

Effort was measured using a 6-item scale from Day et al. (2017), tapping exploration and exploitation aspects of task-focused learning effort relevant to self-regulated learning in complex performance contexts (Hardy et al., 2019a). Example items are “How hard did you try to learn something new in the previous two games?” and “How hard did you try to perform well during the previous two games?” Answers were made on an 11-point Likert scale, with anchors at (0) *Not at all* and (10) *Extremely hard*. Across the 14 sessions, the mean alpha reliability was 0.90 (min. = 0.85, max. = 0.95). While self-report measures have been shown to yield lower criterion-related validity than other more objective measures of effort such as time on task, self-report measures of effort are better able to directly tap into a participants’ task-focused learning effort, with less contamination from construct irrelevant variance (Yeo and Neal, 2004).

#### Task Performance

Using the same formula as Hardy et al. (2019b), task performance scores for each trial were calculated by taking the number of kills (i.e., the number of times that a participant destroyed a computer-controlled bot) divided by the quantity of kills plus player deaths (i.e., the number of times a participant themselves is destroyed), plus player rank (i.e., the participant’s rank relative to the bots within the trial). To increase ease of interpretability, performance scores were multiplied by 100. A single performance score for each session was calculated by taking the average of the two scores for both trials in that specific session.

### Analyses

Discontinuous growth curve modeling was used to model effort and performance scores across acquisition, transition adaptation (TA), and reacquisition adaptation (RA). Using this modeling technique allowed scores following the task change (i.e., post-change period; reacquisition) to be compared to scores prior to the task change (i.e., pre-change period; acquisition) (Bliese and Lang, 2016). We used a coding scheme recommended by Bliese and Lang (2016), which is shown in Table 1. Specifically, SA refers to the linear rate of change in scores across all sessions (e.g., performance improvements; decreases in effort). TA models discontinuity with a dummy coded variable indicating when the task change has occurred. In the present study, TA reflects the discontinuity in scores (e.g., expected drop in performance following the unexpected task change), comparing post-change scores to pre-change scores. RA refers to the linear rate of changes in scores following the task change taking into account the linear rate of changes prior to the task change. Quadratic acquisition (SA^2^) and reacquisition (RA^2^) were also included to account for curvilinear change in scores across the pre-change and post-change periods (Lang and Bliese, 2009). It is important to note the coefficients TA and RA as shown in the coding scheme displayed in Table 1 are interpreted relative to SA. The effect of TA reflects a difference in scores after the task change relative to the value predicted by SA immediately following the task change. RA reflects the change in score trends across sessions following the task change relative to the rate of score changes across pre-change sessions. A significant TA effect in the absence of a significant RA effect reflects an overall difference in scores on the outcome variable of interest in post-change sessions relative to pre-change sessions. If the results show significant effects for both TA and RA, conclusions regarding the overall difference in scores between pre- and post-change periods depend on the strength and direction of the RA effect.

**TABLE 1:** Coding scheme of change variables in discontinuous mixed-effects growth models.

Variable	Pre-change period							Post-change period						
Measurement occasion (Session)	1	2	3	4	5	6	7	8	9	10	11	12	13	14
Skill acquisition (SA)	0	1	2	3	4	5	6	7	8	9	10	11	12	13
Transition adaptation (TA)	0	0	0	0	0	0	0	1	1	1	1	1	1	1
Reacquisition adaptation (RA)	0	0	0	0	0	0	0	0	1	2	3	4	5	6
Quadratic skill acquisition (SA^2^)	0	1	4	9	16	25	36	36	36	36	36	36	36	36
Quadratic reacquisition adaptation (RA^2^)	0	0	0	0	0	0	0	0	1	4	9	16	25	36

In analyses with effort as the outcome variable, the main effects of spin and pulse were used to test Hypothesis 1. SA × spin and SA × pulse interactions were examined to test Hypothesis 2. TA × spin and TA × pulse interactions were examined to test Hypothesis 4, while RA × spin and RA × pulse were examined to test Hypothesis 5. In the analyses with performance as the outcome variable, the spin × effort and pulse × effort interactions were examined to test Hypothesis 3. Three-way interactions involving spin and pulse with effort and the TA and RA interactions (e.g., TA × spin × effort; RA × spin × effort) were examined to test Hypothesis 6. The nlme package in R, an open source software, was used to conduct the discontinuous mixed-effects growth modeling and analyses (Pinheiro et al., 2016). Conclusions regarding support for the hypotheses were made by examining the statistical significance of effects in relation to improved model fit using log-likelihood values (higher values indicating better fit).

## Results

**FIGURE 2:**
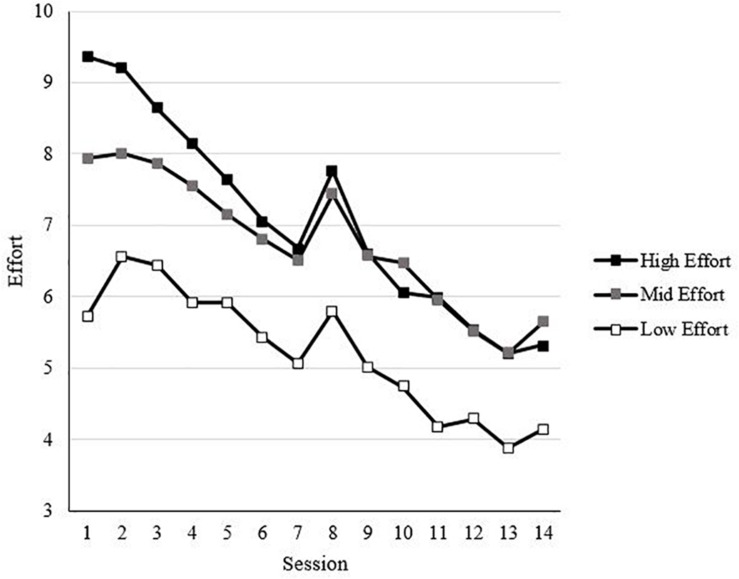
Effort trends across sessions by Session 1 tertiles.

**FIGURE 3:**
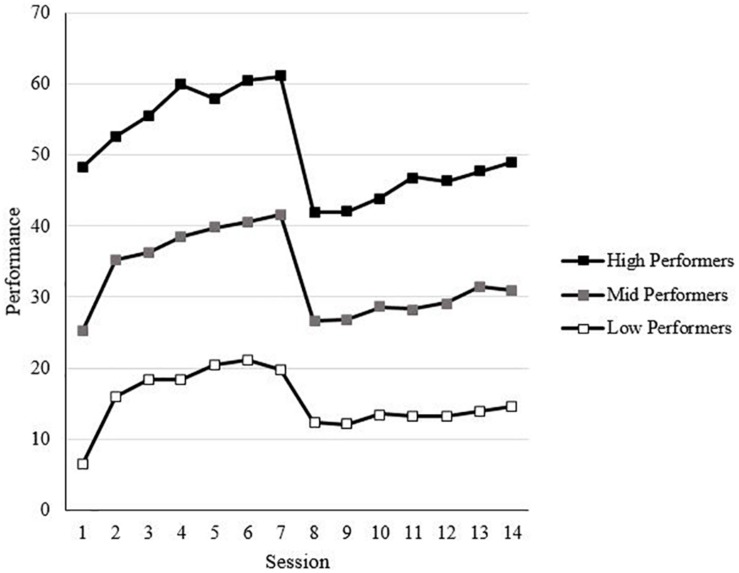
Performance trends across sessions by Session 1 tertiles.

Table 2 displays the descriptive statistics, internal consistency reliabilities, and correlations for all the study variables, along with scores averaged across all sessions for performance and effort. Spin (i.e., within-person fluctuations in affect pleasantness and activation potential) was significantly, negatively correlated with emotional stability (*r* = −0.23, *p* < 0.01), while affect pulse (i.e., within-person fluctuations in affect intensity) was not significantly correlated with emotional stability (*r* = −0.09, *ns*). Both affect spin and affect pulse were correlated with valence variability (*r*s = 0.48 and 0.31, respectively, *p*s < 0.01) and activation variability (*r*s = 0.72 and 0.46, respectively, *p*s < 0.01). Affect spin (*r* = −0.09, *ns*) and affect pulse (*r* = −0.03, *ns*) were not significantly correlated with effort. Affect spin (*r* = −0.16, *p* < 0.05) and affect pulse (*r* = −0.23, *p* < 0.01) were significantly, negatively correlated with performance. Effort was significantly correlated with performance (*r* = 0.18, *p* < 0.05). The trends for effort and performance across sessions are shown in Figures 2, 3, respectively.

**TABLE 2:** Descriptive statistics and correlations.

Variable	*M*	*SD*	1	2	3	4	5	6	7	8	9	10	11	12	13	14
1. Gender^1^	–	–														
2. ACT	26.79	4.09	−0.19**													
3. Video game experience^2^	0.00	1.00	−0.55*	0.16	(0.72)											
4. Openness	6.43	0.88	0.02	0.13^†^	–0.02	(0.75)										
5. Conscientiousness	6.25	0.98	–0.08	−0.11^†^	0.05	0.37**	(0.85)									
6. Extraversion	5.60	1.16	0.03	–0.00	–0.03	0.26**	0.11^†^	(0.89)								
7. Agreeableness	6.86	0.94	0.06	−0.30**	–0.03	0.21**	0.38**	0.13^†^	(0.91)							
8. Emotional stability	5.26	1.06	−0.20*	0.07	0.14*	–0.00	0.24**	0.12^†^	0.22**	(0.85)						
9. Valence variability	3.32	1.51	0.02	−0.12^†^	0.06	0.13^†^	0.00	0.04	0.11	–0.09						
10. Activation variability	2.47	1.03	0.06	–0.01	–0.08	0.16*	–0.10	–0.00	0.02	−0.16*	0.41**					
11. Affect spin	0.83	0.51	0.04	–0.02	−0.14*	0.04	–0.11	–0.06	−0.13^†^	−0.23**	0.48**	0.31**				
12. Affect pulse	2.49	0.94	0.11^†^	−0.21**	–0.05	0.07	–0.05	0.08	0.19**	–0.09	0.72**	0.46**	0.19**			
13. Effort^3^	6.37	1.91	–0.11	–0.01	0.12^†^	0.05	0.16*	0.13^†^	0.08	0.08	0.03	−0.15*	–0.10	–0.04	(0.90)^3^	
14. Performance^3^	32.91	16.96	−0.74**	0.38**	0.63**	0.02	0.08	−0.11^†^	−0.14^†^	0.18*	–0.10	–0.05	−0.16*	−0.23**	0.18*	(0.84)^3^

Diagonal values are coefficient alpha reliabilities. Performance ICC = 0.72. Effort ICC = 0.48. ^1^Gender is a dichotomous variable: 0 = male, 1 = female. ^2^Video game experience was a standardized composite. ^3^Mean alpha across 14 sessions. N = 214. ^†^p < 0.10, *p < 0.05, **p < 0.01, two-tailed.

### Effort

#### Growth Trends

A series of models was first tested following suggestions of Bliese and Lang (2016). We began by testing the basic growth model. Specifically, in Step 1, we tested the effect for each of the time variables included in the equation below (see Model 1 of Table 3):

**TABLE 3:** Discontinuous growth models of effort as a function of affect variability.

	Model 1		Model 2	
Variable	*B*	*SE*	*B*	*SE*
Intercept, γ_00_	7.80**	0.12	7.90**	0.15
Skill acquisition (SA), γ_10_	–0.04	0.07	–0.04	0.07
Transition acquisition (TA), γ_20_	0.98**	0.16	0.98**	0.16
Reacquisition adaptation (RA), γ_30_	−0.76**	0.09	−0.76**	0.09
Quadratic skill acquisition (SA2), γ_40_	−0.04**	0.07	−0.04**	0.01
Quadratic skill reacquisition (RA2), γ_50_	0.08**	0.01	0.08**	0.01
Gender, γ_01_			–0.24	0.24
ACT/SAT, γ_02_			–0.02	0.03
Video game experience (VGE), γ_03_			0.19	0.12
Openness, γ_04_			–0.06	0.13
Conscientiousness, γ_05_			0.25*	0.12
Extraversion, γ_06_			0.20*	0.09
Agreeableness, γ_07_			0.04	0.12
Emotional stability, γ_08_			0.14	0.10
Valence variability, γ_09_			0.30**	0.07
Activation variability, γ_0 10_			−0.25**	0.11
Log-likelihood	−5419.46		−5410.20	

N = 214. *p < 0.05, **p < 0.01, two-tailed.

$$Y_{ij}\;=\;\gamma_{00}\;+\;\gamma_{10}SA\;+\;\gamma_{20}TA\;+\;\gamma_{30}RA\;+\;\gamma_{40}SA^{2}\;+\;\gamma_{50}RA^{2}\;+\;\varepsilon_{ij}$$

As a reminder, SA refers to skill acquisition, specifically linear growth trends across all sessions. TA refers to transition adaptation, specifically the discontinuous change in scores after the changes in task demands (i.e., from Session 7 to 8). RA refers to reacquisition adaptation, specifically the difference in linear growth in post-change sessions relative to the linear growth in pre-change trials. SA^2^ and RA^2^ are included to account for possible curvilinear growth trends.

Although there was no significant SA effect for effort [*t*(2777) = −0.56, *B* = −0.04, *ns*], there was a significant, negative quadratic SA (SA^2^) effect [*t*(2777) = −4.25, *B* = −0.04, *p* < 0.01], showing a decrease in effort scores in pre-change that accelerated across sessions. However, the results also showed a statistically significant, positive TA effect [*t*(2777) = 6.32, *B* = 0.98, *p* < 0.01], as well as a statistically significant negative RA effect [*t*(2777) = −8.12, *B* = −0.76, *p* < 0.01]. The quadratic trend for skill reacquisition (RA^2^) was also statistically significant, but positive [*t*(2777) = 7.73, *B* = 0.08, *p* < 0.01]. This combination of TA, RA, and RA^2^ effects reflects how effort levels dramatically increased after the task change with steady decreases following until an increase in the final session.

In Step 2, the covariates were included (see Model 2 of Table 3). Extraversion [*t*(203) = 2.20, *B* = 0.20, *p* < 0.05], conscientiousness [*t*(203) = 2.20, *B* = 0.25, *p* < 0.05], and valence variability [*t*(203) = 4.16, *B* = 0.30, *p* < 0.01] all showed positive, statistically significant effects, with higher levels of extraversion, conscientiousness, and valence variability associated with higher levels of effort. Activation variability [*t*(203) = −2.34, *B* = −0.25, *p* < 0.05] showed a negative, statistically significant effect, with higher levels of activation variability associated with lower levels of effort. No other covariate yielded a statistically significant effect.

#### Effects of Affect Spin and Pulse

In Step 3, the main effects of spin and pulse on effort were included (see Model 3 of Table 4), neither of which yielded statistically significant effects at this point. Hypotheses 1a and 1b proposed that higher levels of (a) affect spin and (b) affect pulse would be associated with lower initial effort. Thus, the results did not support Hypotheses 1a or 1b.

**TABLE 4:** Discontinuous growth models of effort as a function of affect variability.

	Model 3		Model 4		Model 5	
Variable	*B*	*SE*	*B*	*SE*	*B*	*SE*
Spin, γ_0 11_	−0.20	0.24	–0.08	0.25	–0.11	0.25
Pulse, γ_0 12_	−0.03	0.17	0.08	0.17	0.09	0.17
SA × spin, γ_1 11_			−0.06*	0.03	–0.01	0.06
SA × pulse, γ_1 12_			−0.05**	0.02	−0.06^†^	0.03
TA × spin, γ_2 11_					−0.54^†^	0.30
TA × pulse, γ_2 12_					–0.07	0.16
RA × spin, γ_3 11_					0.02	0.09
RA × pulse, γ_3 12_					0.04	0.05
Log-likelihood	−5411.27		−5407.65		−5410.06	

SA, skill acquisition; TA, transition adaptation; RA, reacquisition adaptation. Across Models 3–5, effects for the growth terms and covariates were substantively unchanged from Model 2 shown in Table 3. N = 214. ^†^p < 0.10, *p < 0.05, **p < 0.01, two-tailed.

**FIGURE 4:**
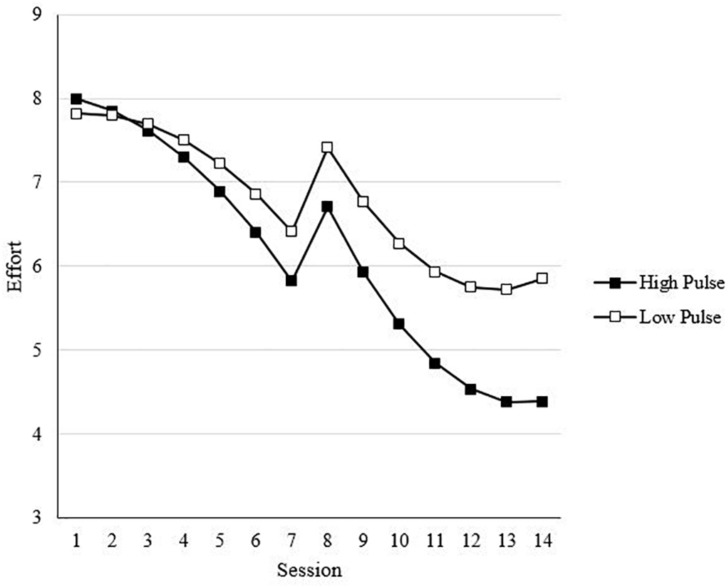
Effect of affect pulse on effect across sessions. Values are predicted scores from model estimates. High/low affect pulse = ±1 standard deviation.

In Step 4, interactions between affect spin and affect pulse with the linear trend for effort (SA) were included (see Model 4 of Table 4). Hypotheses 2a and 2b proposed that higher levels of (a) affect spin and (b) affect pulse would be associated with lower sustained effort. Supporting Hypothesis 2a, there was a statistically significant, negative interaction between affect spin and the SA effort trend [*t*(2775) = −2.08, *B* = −0.06, *p* < 0.05]. Specifically, there was a greater decline in effort for individuals higher in affect spin. A statistically significant, negative interaction with the SA effort trend was also found for affect pulse [*t*(2775) = −3.54, *B* = −0.05, *p* < 0.01], supporting Hypothesis 2b. Moreover, results of the log-likelihood values showed improved fit for this model relative to previous models. However, because this step does not include TA and RA interactions with affect spin and pulse, this lower sustained effort for those higher in affect spin and pulse occurred regardless of the manipulation of task changes. Figure 4 illustrates the trend for pulse. The trend for spin is further discussed below.

**FIGURE 5:**
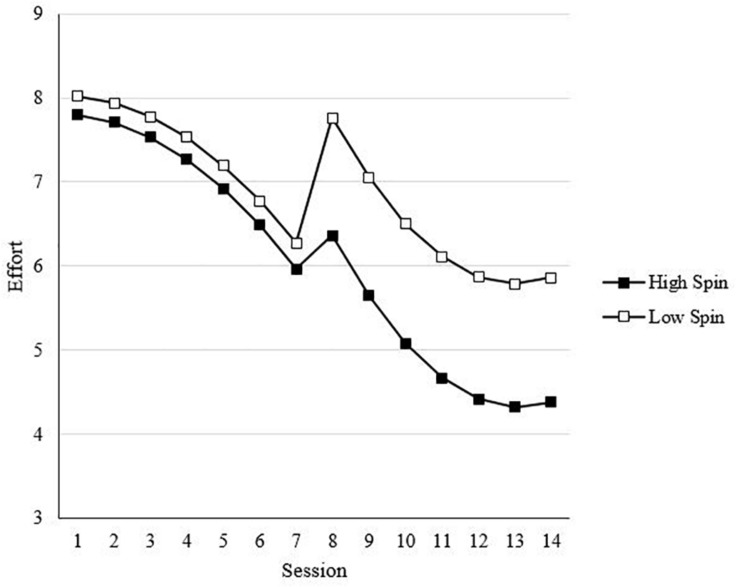
Effect of affect spin on effort across sessions. Values are predicted scores from model estimates. High/low affect spin = ±1 standard deviation.

In the final step, Step 5, interactions involving affect spin and affect pulse with TA and RA were included (see Model 5 of Table 4). This step was used to test Hypotheses 4 and 5. Hypotheses 4a and 4b proposed the negative direct effects of (a) affect spin and (b) affect pulse on overall levels of effort would be stronger in adaptation than acquisition. Hypotheses 5a and 5b proposed that the negative direct effects of (a) affect spin and (b) affect pulse on sustained levels of effort would be stronger in adaptation than acquisition. As shown in Model 5 in Table 4, only the interaction between affect spin and TA was statistically significant. Specifically, consistent with Hypothesis 4a, the negative effect of affect spin was stronger after the transition than prior [*t*(2771) = −1.80, *B* = −0.54, *p* < 0.05, one-tailed]. Figure 5 shows this relationship between affect spin and effort. Altogether, the SA and TA interactions with affect spin showed greater decline in effort across sessions for those high in affect spin as well as overall lower effort in post-change versus pre-change sessions. Although the direction of the TA × spin interaction was consistent with the effect proposed in Hypothesis 4a, it is important to point out that the log-likelihood value in this model did not show improved model fit. Thus, the results showed mixed support for Hypothesis 4a, and no support was shown for Hypotheses 4b, 5a, and 5b.

### Performance

#### Growth Trends

The steps for modeling performance trends followed the same as those for effort. As shown in Model 1 of Table 5, there was a statistically significant positive SA effect [*t*(2777) = 13.78, *B* = 5.47, *p* < 0.01], a statistically significant, negative TA effect [*t*(2777) = −20.31, *B* = −18.88, *p* < 0.01], and a statistically significant, negative RA effect [*t*(2777) = −8.52, *B* = −4.66, *p* < 0.01]. These effects together indicate that, across pre-change sessions, performance levels increased. However, after the task change, performance levels dropped markedly, and, although performance levels again began to rise, the rate of increase was significantly lower than that of the pre-change rate. SA^2^ was significant [*t*(2777) = −9.17, *B* = −0.57, *p* < 0.01], which indicates that increases in performance decelerated across sessions. RA^2^, however, was not significant and therefore was not included in any further model tests.

**TABLE 5:** Discontinuous growth models of performance.

	Model 1		Model 2	
Variable	*B*	*SE*	*B*	*SE*
Intercept, γ_00_	27.70**	1.26	34.75**	1.02
Skill acquisition (SA), γ_10_	5.47**	0.40	5.47**	0.40
Transition acquisition (TA), γ_20_	−18.88**	0.93	−18.89**	0.89
Reacquisition adaptation (RA), γ_30_	−4.66**	0.55	−4.66**	0.41
Quadratic skill acquisition (SA^2^), γ_40_	−0.57**	0.06	−0.57**	0.06
Quadratic skill reacquisition (RA^2^), γ_50_	0.00	0.06		
Gender, γ_01_			−17.00**	1.58
ACT/SAT, γ_02_			0.85**	0.17
Video game experience (VGE), γ_03_			5.02**	0.77
Openness, γ_04_			0.18	0.85
Conscientiousness, γ_05_			1.30^†^	0.77
Extraversion, γ_06_			−1.29*	0.57
Agreeableness, γ_07_			–0.96	0.79
Emotional stability, γ_08_			0.43	0.66
Valence variability, γ_09_			−1.14*	0.47
Activation variability, γ_0 10_			1.03	0.70
Log-likelihood	−11097.28		−10979.94	

N = 214. ^†^p < 0.10, *p < 0.05, **p < 0.01, two-tailed.

In Step 2, the covariates were included (see Model 2 of Table 5). The main effects of ACT [*t*(203) = 4.88, *B* = 0.85, *p* < 0.01] and videogame experience [*t*(203) = 6.50, *B* = 5.02, *p* < 0.01] were both positive and statistically significant, meaning that higher ACT scores and prior video game experience were associated with higher performance scores. Additionally, the main effects of gender [*t*(203) = −10.76, *B* = −16.97, *p* < 0.01], extraversion [*t*(203) = −2.26, *B* = −1.29, *p* < 0.05], and valence variability [*t*(203) = −2.42, *B* = −1.14, *p* < 0.05] were negative and statistically significant, indicating that females exhibited lower levels of performance than did males, and that those with higher levels of extraversion and valence variability had lower performance scores. No other covariate yielded a statistically significant effect.

#### Effects of Affect Spin and Pulse

In Step 3, the main effects of affect spin and affect pulse on performance were included (see Model 3 of Table 6). Although not hypothesized, the main effect of affect spin showed a statistically significant, negative effect [*t*(201) = −2.57, *B* = −4.02, *p* < 0.05], as did affect pulse [*t*(201) = −3.09, *B* = −3.38, *p* < 0.01]. Those with higher affect spin and pulse had lower performance scores. Results of the log-likelihood values showed improved fit for this model relative to previous models.

**TABLE 6:** Discontinuous growth models of performance as a function of affect variability.

	Model 3		Model 4		Model 5		Model 6	
Variable	*B*	*SE*	*B*	*SE*	*B*	*SE*	*B*	*SE*
Spin, γ_0 11_	−4.02*	1.57	−3.88**	1.56	–2.79	1.74	–2.83	1.74
Pulse, γ_0 12_	−3.38**	1.09	−3.22**	1.09	−4.27**	1.18	−4.23**	1.19
Effort, γ_0 13_			0.66^†^	0.34	–0.12	0.39	–0.14	0.39
Spin × effort, γ_0 14_					0.06	0.65	0.23	0.76
Pulse × effort, γ_0 15_					–0.05	0.33	0.07	0.38
SA × spin, γ_1 11_					–0.30	0.28	–0.30	0.28
SA × pulse, γ_1 12_					0.36*	0.15	0.36*	0.15
SA × effort, γ_1 13_					0.19**	0.07	0.18**	0.07
TA × affect, γ_2 11_					1.13	1.79	1.16	1.78
TA × pulse, γ_2 12_					–0.53	0.96	–0.59	0.97
TA × effort, γ_2 13_					–0.38	0.47	–0.28	0.46
RA × spin, γ_3 11_					–0.03	0.35	–0.04	0.35
RA × pulse, γ_3 12_					−0.57**	0.19	−0.56**	0.19
RA × effort, γ_3 13_					–0.03	0.09	–0.05	0.09
SA × spin × effort, γ_1 14_							0.15	0.14
SA × pulse × effort, γ_1 15_							0.01	0.07
TA × spin × effort, γ_2 14_							−2.20**	0.92
TA × pulse × effort, γ_2 15_							–0.43	0.45
RA × spin × effort, γ_3 14_							0.23	0.18
RA × pulse × effort, γ_3 15_							0.06	0.09
Log-likelihood	−10971.66		−10970.12		−10969.82		−10956.96	

SA, skill acquisition; TA, transition adaptation; RA, reacquisition adaptation. Across Models 3–6, effects for the growth terms and covariates were substantively unchanged from Model 2 shown in Table 5. N = 214. ^†^p < 0.10, *p < 0.05, **p < 0.01, two-tailed.

**FIGURE 6:**
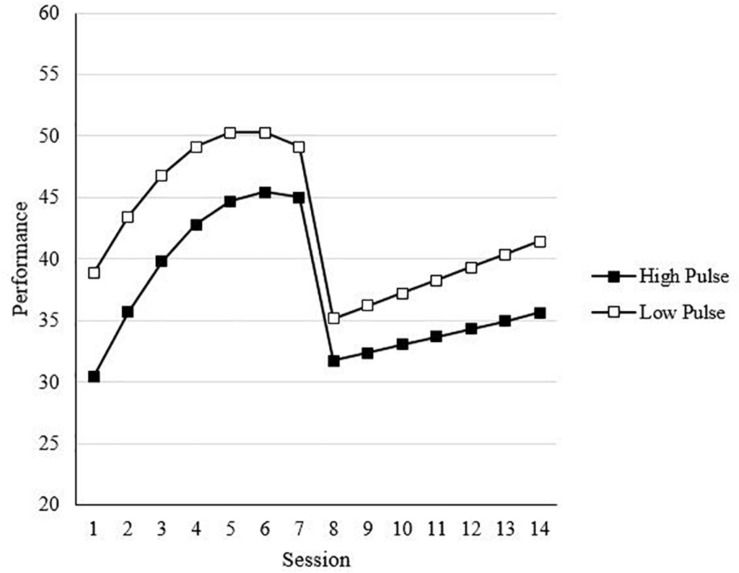
Effect of affect pulse on performance across sessions. Values are predicted scores from model estimates. High/low affect pulse = ±1 standard deviation.

In Step 4, which showed an improvement in model fit, the main effect of effort was included (see Model 4 of Table 6). As one would expect, effort was positively related to performance [*t*(200) = 1.96, *B* = 0.66, *p* < 0.05, one-tailed]. In Step 5, all two-way interactions were included. This step was used to test Hypotheses 3a and 3b, which proposed that affect spin and pulse would moderate the effects of overall effort on performance such that the positive effects of overall effort will be lower for individuals higher on (a) affect spin and (b) affect pulse. As shown in Model 5 of Table 6, neither of the effort interactions involving affect spin and affect pulse were statistically significant. Thus, the results did not support Hypotheses 3a and 3b. Model 5 also included the two-way interactions between affect spin, affect pulse, and effort with SA, TA, and RA. Although no hypotheses were made regarding these interactions, statistically significant interactions were found for both affect pulse and effort (see Model 5 of Table 6). For affect pulse, there was a statistically significant, positive interaction involving SA [*t*(2769) = 2.47, *B* = 0.36, *p* < 0.05], and a statistically significant, negative interaction with RA [*t*(2769) = −3.03, *B* = −0.57, *p* < 0.01]. Figure 6 shows what these interactions involving affect pulse and the growth trends look like, specifically showing that the negative main effect of affect pulse [*t*(200) = −3.24, *B* = −2.72, *p* < 0.05] in Model 5 becomes smaller later in SA (i.e., pre-change sessions), but in post-change, the negative effect of affect pulse becomes stronger later in RA (i.e., post-change sessions). For effort, there was a positive SA interaction [*t*(2769) = 2.63, *B* = 0.19, *p* < 0.01], indicating a stronger positive effect of effort later in SA. It is important to acknowledge that the results of the log-likelihood values showed improved fit for this model relative to previous models.

**FIGURE 7:**
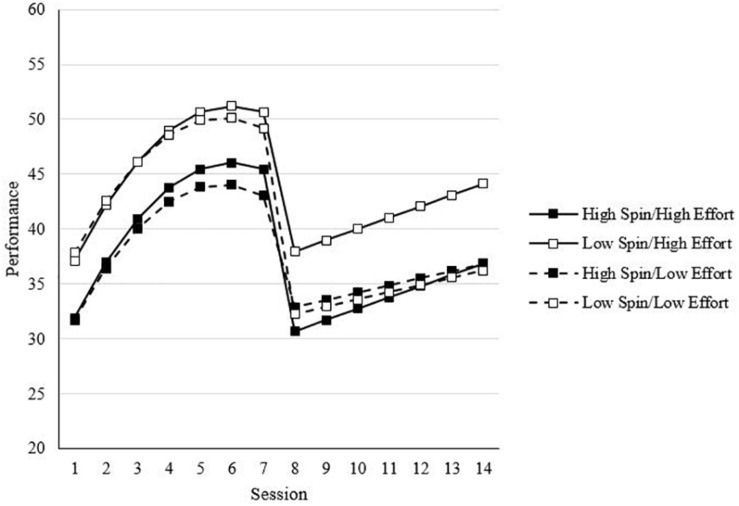
Effect of affect spin on performance across sessions. Values are predicted scores from model estimates. High/low affect spin = ±1 standard deviation.

In the final step, we included three-way interactions between (1) affect spin and affect pulse, (2) effort, and (3) SA, TA, and RA. This model tested Hypotheses 6a and 6b, which proposed that the negative moderation effect of (a) affect spin and (b) affect pulse on the effort-performance relationship would be stronger in adaptation than acquisition. The only statistically significant interaction found was between affect spin, effort, and TA [*t*(2763) -2.39, *B* = −2.20, *p* < 0.05]. While the results did not support Hypothesis 6b, they did support Hypothesis 6a, with stronger negative moderation effects of affect spin found after the task changes (i.e., TA). As shown in Figure 7, after the task changes, there was a positive effect of effort for individuals low in affect spin, but there was not a positive effect of effort for individuals high in affect spin. In other words, after the task change, effort was beneficial to performance only for individuals low in affect spin. Also shown in Figure 7 before the transition, effort yielded small beneficial effects, regardless of affect spin. Results of the log-likelihood values showed improved fit for this model relative to all previous models.

### Ancillary Analyses

Ancillary analyses were conducted to further examine the distinctiveness of affect spin and pulse vis-à-vis emotional stability. Although our primary analyses showed that affect spin and pulse accounted for additional variance in effort and performance beyond emotional stability, these ancillary analyses were conducted to shed more light on whether affect spin and pulse should be considered as meaningfully distinct from or as components of emotional stability. The ancillary analyses followed the same steps as the primary analyses, however, the predictor variables of spin and pulse were removed, and emotional stability was substituted in their place. Thus, the direct effects of emotional stability on effort and performance were tested, along with all the same interactions previously tested for spin and pulse involving effort and the growth trends (i.e., SA, TA, and RA).

The results for emotional stability followed several key patterns as compared to affect spin (although in the opposite direction, given that emotional stability and affect spin were negatively correlated; *r* = −0.23, *p* < 0.01). Specifically, emotional stability showed a positive interaction with TA on effort [*t*(2774) = 2.15, *B* = 0.30, *p* < 0.05], and a positive interaction with TA and effort on performance [*t*(2769) = 2.04, *B* = 0.82, *p* < 0.05]. However, results of the log-likelihood values did not show improved fit for the models including these interactions. In contrast to the results for affect spin, emotional stability did not yield a significant interaction with SA on effort [*t*(2776) = −1.00, *B* = −0.01, *ns*] or a main effect on performance [*t*(203) = 0.65, *B* = 0.43, *ns*]. Altogether the results suggest that affect spin could be a component of emotional stability, but nevertheless the results indicate affect spin does provide additional insight into acquisition and adaptability above and beyond emotional stability.

The results for emotional stability showed no effects that were similar to those for pulse. Again, emotional stability did not interact with SA on effort nor did it yield a main effect on performance like pulse. Furthermore, it did not yield a significant SA interaction on performance [*t*(2772) = −0.20, *B* = −0.03, *ns*] or a significant RA interaction on performance [*t*(2772) = 0.43, *B* = 0.07, *ns*] like pulse did. Also, affect pulse and emotional stability were not correlated with each other (*r* = −0.09, *ns*). Thus, the findings of the present study indicate that affect pulse is meaningfully distinct from emotional stability.

## Discussion

The broad aim of this lab study was to explore the construct of adaptability as a constellation of individual differences involving sustained effort in the face of unexpected task changes (Bell and Kozlowski, 2008; Niessen and Jimmieson, 2016), specifically the role of dynamic personality factors that extend beyond traditional measures of the Big Five. Using a repeated measures design, the present study examined affect variability—both spin and pulse—in the context of complex task learning with the aim of better understanding the non-cognitive traits that impact people’s capacity to be successful when learning new tasks and adapting to changes in task demands. In doing so, this study addressed the popular advice of “keep calm and carry on.”

Several hypotheses regarding spin and pulse were supported. Both aspects of affect variability undermined the ability to sustain effort, regardless of any changes in task demands. In addition, affect spin moderated the relationship between effort and performance during adaptation, such that effort was only beneficial during adaptation (i.e., post-change sessions) when learners exerting high effort were also low in affect spin. Although affect pulse did not moderate the effort-performance relationship, it yielded a direct negative association with performance, with this effect being stronger during adaptation. Moreover, these relationships were observed after controlling for emotional stability, the other Big Five personality variables, valence variability, and activation variability, and thus speak to the extent to which spin and pulse are distinct aspects of personality that can provide additional insight to behavioral outcomes.

### Direct Effect on Effort

When examining the effects of affect spin and pulse, two broad theoretical mechanisms were tested. First, it was proposed that spin and pulse would undermine effort, with those higher in spin and pulse not exerting as much initial task-related effort, as well as not sustaining effort levels over time. Results showed that, although affect spin and pulse did not have a negative effect on initial effort, both affect spin and pulse negatively impacted sustained effort, with those high in affect variability not being able to maintain effort levels over time as well as those lower in affect variability. These results provide support for the first theoretical mechanism proposed, indicating that those higher in spin and pulse did not direct as much effort toward the task at hand. Taking into consideration previous research on affect variability, there are several potential explanations for these results. As mentioned earlier, the general profile of those high in affect variability speaks to an increase in reactivity to emotionally charged events, difficulties in adjustment, and a general negative profile that includes negative expectations for the future (Kuppens et al., 2007; Beal et al., 2013). These characteristics in turn can lead to greater strain (Beal et al., 2013), the depletion of cognitive resources via emotion regulation (Park, 2015), and even burnout (Giorgi et al., 2017), all of which hinder the ability to sustain effort over time.

Although this study targeted reactive adaptation, the findings that affect spin and pulse yielded negative SA interactions on effort also speaks to proactive adaptation, such that individuals with high affect variability struggled to maintain the effort levels necessary to successfully continue to improve performance regardless of any changes in task demands. Proactive adaptation involves taking initiative to create new demands and making strides to improve performance irrespective of changes in task demands (Ployhart and Bliese, 2006; Berg et al., 2010; Huang et al., 2014). The negative interactions involving spin and pulse with SA suggests that those high in affect variability are less likely to put forth the effort necessary to recognize the subtle differences in various performance strategies, find more effective strategies, and make the adjustments needed to reach higher levels of performance regardless of any changes in task demands. In this way, adaptability can be considered an important aspect of both SA and adaptation.

### Moderation of the Effort-Performance Relationship

The second theoretical mechanism proposed that spin and pulse would moderate the effort-performance relationship. Although not supported in terms of affect pulse, this theoretical mechanism was supported for affect spin. After the task change, high levels of effort were only beneficial for those low in affect spin. In other words, the effort put forth by those high in affect spin was not helpful to performance. There are several aspects of affect variability that may be related to this moderation effect. First, building upon previous strategies, which is necessary to continue making performance gains, may be difficult for those experiencing a high level of strain, which has been shown to disrupt memory (Dhabhar, 2018). In addition, experiencing a constant shift in emotions likely leads to haphazard learning, directed by emotion rather than a systematic learning strategy. This haphazard learning makes it difficult for individuals to discover and adjust performance strategies.

Although not considered, it was found that affect variability yielded a direct, negative association with performance, meaning that those high in affect spin and pulse had lower performance scores. Along with this main effect, it was found that across acquisition the positive pulse-performance association weakened as the task became proceduralized and less cognitive resources were required to sustain performance (Kanfer and Ackerman, 1989). In contrast, across adaptation the positive pulse-performance association became stronger. Below we discuss how our findings concerning these direct associations could be due to limitations in our measurement of effort. However, we also speculate direct associations may be plausible because variability in emotions might cause learners to use more intentional processing (i.e., explicit learning) rather than incidental processing (i.e., implicit learning), the latter of which has been shown to be more conducive to learning in fast-paced, complex performance contexts such as the one involved in the present study (Lewicki et al., 1992; DeShon and Alexander, 1996).

Combined with the previously mentioned negative impact on effort levels (regardless of task change), these results show that high affect spin and pulse are a hindrance to learning in fast-paced, complex performance contexts, in terms of both acquisition and adaptation. Simply put, low levels of affect spin and pulse are important aspects of adaptability. This clearly speaks to the popular advice, “keep calm and carry on,” as a more even demeanor would be beneficial to sustaining effort over time, thereby increasing performance levels. However, following this advice may prove to be a challenge for those high in affect variability.

Thus, our findings suggest that low affect spin and pulse are important for occupation and performance environments that require an even demeanor, such as those that are fast-paced, emphasize continuous learning, or involve unpredictable changes. In particular, environments that require a lot of autonomous learning may not be suitable for those high affect spin and pulse, because autonomous learning requires a great deal of sustained task attention and emotion control (Kanfer and Ackerman, 1989; Bell and Kozlowski, 2008).

### Distinctiveness of Affect Variability

The present study advances theory in terms of how both affect spin and pulse are important non-cognitive traits that help comprise the construct of adaptability (Baard et al., 2014). Affect spin and pulse uniquely address aspects of personality that are not captured by traditional measures of the Big Five—namely the idea that fluctuations in the expressions of traits should be expected across time (Fleeson and Jayawickreme, 2015). The repeated-measures structure to measuring affect spin and pulse captures individual differences in within-person fluctuations in affect expression, leading to a more holistic understanding of emotional stability and personality more generally. Specifically, this study advances our understanding of affect spin and pulse as important contributors to SA and adaptation, with spin showing patterns consistent with it being a meaningful, yet distinct, aspect of emotional stability and affect pulse being an altogether distinct personality construct (cf. Bolger and Zuckerman, 1995; Kuppens et al., 2007). This finding also speaks to the commonplace advice of “keep calm and carry on.” While it may be beneficial to performance to be able to maintain an even-keeled emotional state, this advice may not be realistic for those high in affect spin and pulse, and effort and performance may suffer as a consequence.

It should be emphasized that pulse as well as spin uniquely explained variance in effort and performance, suggesting that both are important predictors of behavioral outcomes. Foundational research on affect variability concluded that affect spin, but not pulse, was a unique and central contributor to psychological well-being (Kuppens et al., 2007), and consequently later research on affect variability focused on affect spin in lieu of pulse (e.g., Beal et al., 2013; Park, 2015; Clark et al., 2016). Thus, in contrast to much of the burgeoning literature on affect variability, the results of the current study highlight the importance of considering both spin and pulse as meaningful contributors to behavioral outcomes, specifically effort and performance in fast-paced, complex performance environments. As such, and more broadly, it would be beneficial for future empirical research on affect variability to include both spin and pulse in relation to behavioral outcomes to better understand how personality contributes to adjustment and psychological well-being.

### Limitations and Future Research

There are several limitations to this study that should be considered when attempting to interpret and generalize these results. First, the task itself may have impacted the results for several reasons. It is likely that the fast-paced and complex nature of the task may have differentially impacted participants who were high in spin and pulse. Dealing with a fast-paced, complex game with strong perceptual-motor demands may elicit and exacerbate the deleterious effects of spin and pulse not evidenced in other performance contexts, considering that those high in affect variability show a heightened reaction to stressful and emotionally charged events (Beal and Ghandour, 2011). It is also possible that providing monetary bonuses for high performance may have put additional pressure on participants and further amplified the effects of affect variability. Although not a focus of the present study, our results showed clear performance differences between males and females, thus pointing to the possibility that gender may be an additional factor that complicates how the effects of affect variability might be dependent upon the particular demands of the performance context. Therefore, we recommend that future research attempts to replicate the present results across a variety of performance contexts and examine possible gender-based effects in relation to task-based factors.

In addition to the demands of the performance context, effective task strategies were not specifically communicated to the participants as part of their training. This approach to training is qualitatively different from a more proceduralized learning environment where learners are given consistent direction and feedback, and thus the results may not generalize to more proceduralized training. However, there are advantages to training under this less proceduralized style. Research shows that active-learning environments, where learners are more engaged and in control of their learning, lead to better transfer outcomes, especially in terms of adaptive performance (Keith and Wolff, 2015). Thus, the learning environment of this study fits well with the type of learning environments that are thought to better translate into generalizable performance. Nevertheless, future research is needed to examine how the effects of affect variability might be moderated by aspects of the learning environment independent of the influence of task demands. It is possible that more proceduralized learning environments could reduce the emotional reaction experienced by those high in spin and pulse, thus mitigating their harm to effort and performance.

Also of note is the self-report nature of our measure of effort. It is possible that this measure did not fully encompass all aspects of task-based effort. For example, it is likely that participants are unable to fully monitor how much effort they are exerting toward on-task attention, and thus may either over- or underestimate the true amount of effort being exerted. Rather than rely on self-reports, future research could utilize physiological measures such as eye-tracker technology, which has been used in the past to measure on-task attention, under the assumption that people are focusing on what they fixate upon foveally (Duchowski, 2002; Moran et al., 2016). An additional option would be the use of electroencephalogram (EEG) technology. By using EEG to monitor brain states that indicate control and utilization of attention, attention could be more directly measured as opposed to only using self-reports. If the full picture of effort exerted by participants was not captured by the self-report measure, there may have been aspects of the spin and pulse relationships with effort and their moderation of the effort-performance relationship that were not captured in this study. It is also possible that the inability of the self-report measure to truly capture actual effort (versus perceived) was the root of the unexpected direct relationship with affect pulse and performance.

Furthermore, although the hypotheses in this study were based on existing theory and empirical research, the underlying processes by which we proposed spin and pulse would have an effect on effort and performance were not directly tested. Future research should directly measure off-task attention, emotion regulation, and perceived strain to more directly examine the specific processes by which spin and pulse undermine effort and performance. Including such measures would likely provide a better picture of the underlying effects of spin and pulse. For example, self-reports of high levels of effort do not directly capture how some aspects of attention may be directed to non-task-related issues (e.g., worry, self-doubt), however, directly measuring off-task attention and mind wandering would. Future research could also examine the extent to which spin and pulse are related to strategy-switching behaviors, in accordance with the moderation mechanism proposed in this study. A better understanding of the underlying causal processes of spin and pulse may in turn lead to the development of interventions that could help foster learning and adaptive performance for those high in spin and pulse. As such, future research should focus on approaches by which the detrimental effects of spin and pulse might be mitigated, which would provide insight into practical recommendations. In general, future research addressing the extent to which the deleterious effects of spin and pulse on learning and adaptive performance can be mitigated is needed before clear recommendations for practice should be made.

Finally, it is also important to acknowledge the cross-sectional, correlational nature of the present study’s design, which limits the extent to which clear causal conclusions can be made about the effects observed. Scores for affect variability, effort, and performance were taken during the same period of time. Thus, fluctuations in effort and performance may have caused some of the fluctuations in emotion from which the scores for affect spin and pulse were calculated. To get a better sense for the causal effects of spin and pulse as personality traits *per se*, we recommend future studies adopt a predictive design and derive spin and pulse scores from a period of time preceding the time when behavioral outcomes are measured.

## Conclusion

In summary, the current study furthers our understanding of the non-cognitive aspects of adaptability by demonstrating affect spin and pulse are related yet meaningfully distinct aspects of personality that differentially predict effort and performance. In this way, our results show that consistency in emotion, in terms of both type and intensity, is important to maintaining task-focused effort and high performance levels. Accordingly, measurement approaches that address the dynamic aspects of personality not captured by traditional measures are important to understanding human performance, particularly what it means to adapt in fast-paced, complex performance settings. Future research involving different tasks and learning contexts with a focus on testing specific mediating mechanisms underlying performance is needed to further our understanding of the unique effects of affect variability and provide actionable recommendations for improving behavioral outcomes for individuals who find it difficult to “keep calm and carry on.”

## Data Availability Statement

The datasets generated for this study are available on request to the corresponding author.

## Ethics Statement

The studies involving human participants were reviewed and approved by the Institutional Review Board for the Protection of Human Subjects, University of Oklahoma. The patients/participants provided their written informed consent to participate in this study.

## Author Contributions

KR and ED contributed to the conception and design of the work, the analysis and interpretation of data, and the drafting and revising of the work. AJ and JH contributed to the design of the work, the analysis of the data, and the revising of the work.

## Conflict of Interest

The authors declare that the research was conducted in the absence of any commercial or financial relationships that could be construed as a potential conflict of interest.
